# Deciphering Treg cell roles in esophageal squamous cell carcinoma: a comprehensive prognostic and immunotherapeutic analysis

**DOI:** 10.3389/fmolb.2023.1277530

**Published:** 2023-09-28

**Authors:** Pengpeng Zhang, Shiyang Dong, Wei Sun, Wan Zhong, Jingwen Xiong, Xiangjin Gong, Jun Li, Haoran Lin, Yu Zhuang

**Affiliations:** ^1^ Department of Lung Cancer Surgery, Tianjin Medical University Cancer Institute and Hospital, Tianjin, China; ^2^ Department of Thoracic Surgery, The First Affiliated Hospital of Nanjing Medical University, Nanjing, China; ^3^ Department of General Surgery, Fuyang Tumour Hospital, Fuyang, China; ^4^ Department of Thoracic Surgery, The Second Hospital of Nanjing, Nanjing, China; ^5^ Department of General Surgery, The Fourth Affiliated Hospital of Nanjing Medical University, Nanjing, China; ^6^ Department of Sports Rehabilitation, Southwest Medical University, Luzhou, China; ^7^ Department of Thoracic Surgery, Nanjing Chest Hospital, Nanjing, China; ^8^ Afliated Nanjing Brain Hospital, Nanjing Medical University, Nanjing, China

**Keywords:** ESCC, regulatory T cells, immunotherapy, prognosis, signature

## Abstract

**Background:** Esophageal squamous cell carcinoma (ESCC) is a prevalent and aggressive form of cancer that poses significant challenges in terms of prognosis and treatment. Regulatory T cells (Treg cells) have gained attention due to their influential role in immune modulation within the tumor microenvironment (TME). Understanding the intricate interactions between Treg cells and the tumor microenvironment is essential for unraveling the mechanisms underlying ESCC progression and for developing effective prognostic models and immunotherapeutic strategies.

**Methods:** A combination of single-cell RNA sequencing (scRNA-seq) and bulk RNA-seq analysis was utilized to explore the role of Treg cells within the TME of ESCC. The accuracy and applicability of the prognostic model were assessed through multi-dimensional evaluations, encompassing an examination of the model’s performance across various dimensions, such as the mutation landscape, clinical relevance, enrichment analysis, and potential implications for immunotherapy strategies.

**Results:** The pivotal role of the macrophage migration inhibitory factor (MIF) signaling pathway within the ESCC TME was investigated, with a focus on its impact on Treg cells and other subpopulations. Through comprehensive integration of bulk sequencing data, a Treg-associated signature (TAS) was constructed, revealing that ESCC patients with elevated TAS (referred to as high-TAS individuals) experienced significantly improved prognoses. Heightened immune infiltration and increased expression of immune checkpoint markers were observed in high-TAS specimens. The model’s validity was established through the IMvigor210 dataset, demonstrating its robustness in predicting prognosis and responsiveness to immunotherapy. Heightened therapeutic benefits were observed in immune-based interventions for high-TAS ESCC patients. Noteworthy differences in pathway enrichment patterns emerged between high and low-TAS cohorts, highlighting potential avenues for therapeutic exploration. Furthermore, the clinical relevance of key model genes was substantiated by analyzing clinical samples from ten paired tumor and adjacent tissues, revealing differential expression levels.

**Conclusion:** The study established a TAS that enables accurate prediction of patient prognosis and responsiveness to immunotherapy. This achievement holds significant implications for the clinical management of ESCC, offering valuable insights for informed therapeutic interventions.

## 1 Introduction

Esophageal squamous cell carcinoma (ESCC) represents a pressing global health challenge, characterized by its alarming mortality rates and intricate tumor microenvironment (TME). Recent years have witnessed both advances in treatment strategies and the emergence of immunotherapeutic interventions, casting a hopeful light on ESCC management (Yamamoto and Kato, 2022). Immunotherapy, in particular, has garnered significant attention, offering novel avenues for therapeutic exploration. However, a comprehensive understanding of the TME, specifically the intricate involvement of regulatory T (Treg) cells, remains pivotal for optimizing immunotherapy and devising prognostic tools (Zou, , 2006).

In recent years, the mortality rates associated with ESCC have posed significant challenges within the realm of oncology. Despite incremental improvements in therapeutic strategies, ESCC mortality remains distressingly high. The pursuit of novel treatment avenues has culminated in the emergence of immunotherapy, presenting a promising approach to addressing this clinical conundrum (Ozer and Sahin, 2022). Immunotherapy has transformed the landscape of ESCC treatment, shifting the focus from conventional modalities towards harnessing the power of the immune system. Immune checkpoint blockade, particularly targeting programmed cell death protein 1 (PD-1) and programmed death-ligand 1 (PD-L1) interactions, has shown encouraging efficacy in subsets of ESCC patients (Markar, 2022). These therapies unleash the immune system’s potential to recognize and eliminate malignant cells, offering prospects of prolonged survival and improved quality of life. The intricate TME of ESCC is characterized by multifaceted interactions between malignant cells and the surrounding stromal and immune components. Stromal cells, immune infiltrates, cytokines, and extracellular matrix constituents interplay to shape tumor progression, invasion, and therapy resistance. A comprehensive comprehension of this complex milieu is essential for devising tailored therapeutic interventions. Central to the intricate TME of ESCC are Treg cells, which play a dual role in immune homeostasis and suppression. In the context of ESCC, Treg cells create an immunosuppressive niche that fosters tumor immune evasion and growth (Yue et al., 2020). As immunotherapy gains prominence, understanding the interplay between Treg cells and the immune response becomes crucial for circumventing immune resistance and enhancing treatment outcomes.

In pursuit of personalized and precise therapeutics, the integration of scRNA-seq with bulk RNA-seq analysis holds immense potential. By dissecting the heterogeneity of TME cellular constituents, including Treg cells, a comprehensive understanding of their functional diversity and impact on treatment response can be attained. Constructing a Treg-associated signature (TAS) through this integrative approach offers a promising avenue for predicting patient prognosis and stratifying individuals likely to benefit from immunotherapy.

## 2 Methods

### 2.1 Dataset source

The utilization of the Cancer Genome Atlas (TCGA) database (https://portal.gdc.cancer.gov/) facilitated the acquisition of bulk RNA-seq data, mutation data, and clinical characteristics pertaining to patients diagnosed with ESCC. From the Gene Expression Omnibus (GEO) database (http://www.ncbi.nlm.nih.gov/geo/), a scRNA-seq dataset (GSE188900) encompassing tissues from 6 ESCC patients (Pan et al., 2022), comprising 7 surgically resected tumor tissue samples and 1 normal tissue sample, was obtained. Inclusion of external validation cohorts (GSE53624, iMvigor 210) in the analysis was also undertaken. To ensure uniformity and comparability of data, the expression data was converted into the transcripts per million (TPM) format. Mitigation of any potential batch effects was performed using the “combat” function within the “sva” R package (Zhang et al., 2020). Furthermore, the TCGA database supplied bulk sequencing data, mutation data, and clinical details of ESCC patients. All of which were log2-transformed to attain a standardized data format prior to commencement of the analysis.

### 2.2 scRNA-seq data analysis

The initial steps of cell clustering and dimension reduction were carried out employing the R package “Seurat” (Cao et al., 2022; Zhang et al., 2023). Cells were excluded if their gene expression encompassed over 5,000 genes or fewer than 300 genes, or if the proportion of unique molecular identifiers (UMIs) originating from the mitochondrial genome surpassed 10%. The dataset’s dimensionality underwent reduction through the application of principal component analysis (PCA) to the subset of variably expressed genes (Ren et al., 2023; Wang et al., 2023). Following this, cluster analysis was executed via utilization of the “FindClusters” function, considering the top 20 PCA components. After iterative adjustments, a resolution of 0.8 was designated to enhance the distinction among subgroups effectively. Subsequent annotation of the resultant two-dimensional representation of cell clusters was performed using canonical marker genes, enabling the identification of established biological cell types. To ascertain the marker genes associated with cell clusters, the Seurat “FindAllMarkers” function was harnessed to conduct comparisons between cells within a specific cluster and cells encompassing all other clusters. For the inference of communication networks between cell subpopulations, the “cellchat” R package (Jin et al., 2021) was employed (Liu et al., 2023).

### 2.3 Building a high-performance TAS

Prognostic key genes were identified through the implementation of univariate Cox regression and lasso regression analyses (Cui et al., 2023). Subsequent to this, a refinement process was executed to select the genes and ascertain their corresponding coefficients utilizing multivariate Cox regression. The risk score calculation for patients diagnosed with ESCC was conducted according to the subsequent formula: Risk score = $\sum_{k=1}^{n}\text{Coef}\_k\times \text{Expr}\_k$ , wherein Coef_k represents the abbreviation for regression coefficients, and Expr_k signifies the expression level of prognostic model genes. The computation of the risk score was extended to the ESCC patients present within the dataset, resulting in their stratification into high- and low-TAS groups based on the median risk score. The evaluation of the model’s predictive efficacy was carried out by employing receiver operating characteristic (ROC) curves, with an area under the curve (AUC) value surpassing 0.65 indicative of exceptional performance. Additionally, the utilization of PCA facilitated the visual representation of the distribution of patients across distinct risk groups.

### 2.4 Clinical correlation

We integrated clinical features, risk stratification, and gene expression levels into our analysis, employing the “pheatmap” package to craft informative heatmaps. Subsequently, we grouped patients according to their clinical attributes, aiming to explore potential associations between clinical characteristics and the stability of our model. This approach allows us to comprehensively assess how clinical factors may impact the robustness and reliability of our model. By doing so, we gain valuable insights into the potential real-world applicability and generalizability of our findings in different clinical contexts.

### 2.5 Enrichment analysis

We employed GSVA, a non-parametric method, to deduce the activity levels of biological pathways or gene sets across individual samples. GSVA leverages the complete expression profile of each sample, comparing it against predefined gene sets to quantify the enrichment of specific pathways. This approach offers a holistic perspective on variations in pathway activity within our dataset (Subramanian et al., 2005). In parallel, we utilized GSEA, a widely recognized enrichment analysis tool, to assess whether predefined gene sets displayed statistically significant differences in their expression patterns between two predefined biological states. GSEA achieves this by ranking genes based on their expression changes across various conditions and then evaluating the enrichment of gene sets within these ranked lists. Consequently, GSEA unveils pertinent biological pathways associated with the specific experimental or clinical context under investigation. By combining GSVA and GSEA, we facilitated a more in-depth exploration of pathway activities and their biological significance within our dataset. This synergistic approach enhanced our comprehension of the underlying molecular mechanisms, shedding light on potential implications for the phenomenon under study.

### 2.6 Mutation analysis

Through utilization of the “maftools” R package (Mayakonda et al., 2018), an in-depth examination was conducted concerning the frequency and distribution of somatic mutations spanning diverse genes. This analytical endeavor facilitated the identification of genes exhibiting noteworthy mutation rates, which, in turn, hold the potential to assume pivotal roles in the pathogenesis of ESCC. Moreover, the inherent capabilities of the package enabled the visualization of patterns associated with mutation co-occurrence and mutual exclusivity. This visual representation cast illuminating insights onto plausible synergistic or antagonistic interactions present amid distinct genetic alterations. In parallel, TCGA-ESCC patients underwent a stratification process resulting in their categorization into four discrete groups predicated upon both median risk score and median tumor mutational burden (TMB). A subsequent comparative analysis was undertaken, scrutinizing the survival disparities among these groups based upon their respective median risk scores and TMB values.

### 2.7 The TME and immunotherapy

Seven distinct immune infiltration algorithms were harnessed to rigorously assess the immune cell composition, facilitated through access to the comprehensive resources provided by the timer 2.0 database (http://timer.comp-genomics.org/). Employing this approach, a comprehensive evaluation of the immune landscape within the context of the study was achieved. Subsequently, to convey the intricate variances in immune cell infiltration across diverse risk groups, heatmaps were employed as an effective visual representation, thereby elucidating the nuances within immune cell populations. Furthermore, the quantification of immunological scores, stromal scores, and ESTIMATE scores for patients diagnosed with ESCC was meticulously carried out using the specialized functionalities afforded by the “estimate” R package (Yoshihara et al., 2013). This strategic deployment empowered a robust assessment of the TME and its potential implications. In the pursuit of identifying potentially efficacious chemotherapeutic agents among the various risk groups, the predictive capabilities of the “oncoPredict” R package (Maeser et al., 2021) were harnessed. By leveraging this tool, an insightful prognostication of suitable therapeutic interventions was facilitated, contributing to a more informed treatment strategy.

### 2.8 qRT-PCR

Ethical approval was secured from the Medical Ethics Committee (No. 2SRFA-005) of the First Affiliated Hospital of Nanjing Medical University for the procurement of tissue specimens. These specimens, encompassing tumor tissue (T) and precancerous tissue (N), sourced from ESCC patients who underwent tumor resection, were diligently preserved at −80°C. Total RNA extraction from ESCC tissues was executed employing the TRIzol reagent, a product of Thermo Fisher Scientific headquartered in Waltham, MA, United States. The subsequent cDNA synthesis followed the manufacturer’s guidelines, utilizing the RevertAid™ First Strand cDNA Synthesis Kit, also provided by Thermo Fisher Scientific. The qRT-PCR analysis transpired utilizing the StepOne Real-Time PCR system, an instrument likewise manufactured by Thermo Fisher Scientific. For amplification, the SYBR Green PCR kit from Takara Bio in Otsu, Japan, was employed. The quantification of relative gene expression levels was achieved via the 2^-△△CT^ method.

### 2.9 Statistical methods

We utilized R version 4.2.0 to conduct a comprehensive range of statistical analyses and data processing procedures. To establish statistical significance, we employed Kaplan-Meier curves for survival analysis, complemented by the application of the log-rank test. The “survminer” R package was instrumental in generating the complete set of survival curves. To visually represent data and explore patterns, we skillfully constructed informative heatmaps using the specialized functionalities offered by the “pheatmap” R package. For variables that followed a normal distribution, we quantified quantitative differences through two-tailed t-tests or one-way analysis of variance, depending on the context. In cases where the data exhibited a non-normal distribution, we applied either the Wilcoxon test or the Kruskal-Wallis test to assess differences. It is essential to emphasize that all statistical analyses were conducted within the R programming environment, adhering to a stringent *p*-value threshold of less than 0.05. This threshold served as the critical criterion for identifying and interpreting statistical significance, ensuring the robustness of our findings.

## 3 Results

### 3.1 The analysis of scRNA profiling in ESCC

**FIGURE 1:**
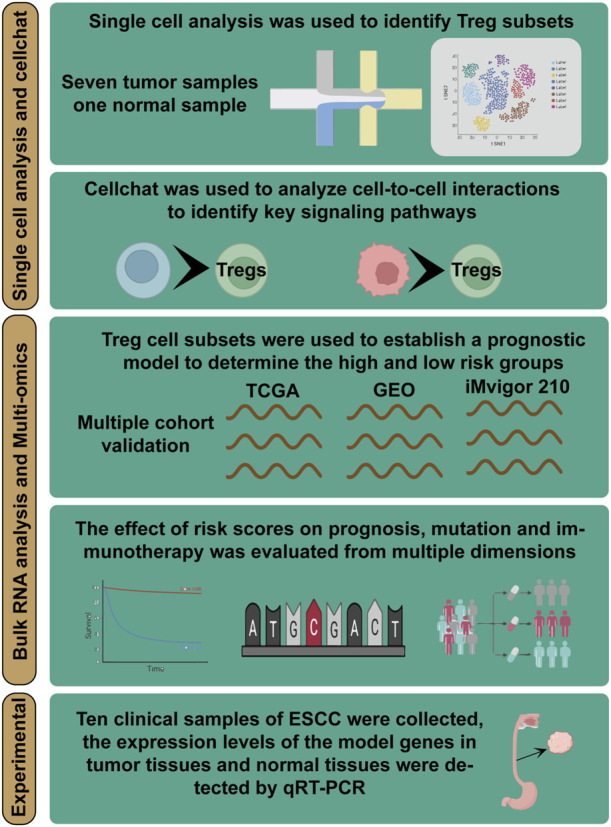
Overall flowchart of all analyses. Integrating single-cell and bulk transcriptome analyses for a comprehensive exploration of Treg cells function in ESCC and building a TAS for predicting the prognosis and immunotherapy of ESCC patients—a multi-omics analysis.

**FIGURE 2:**
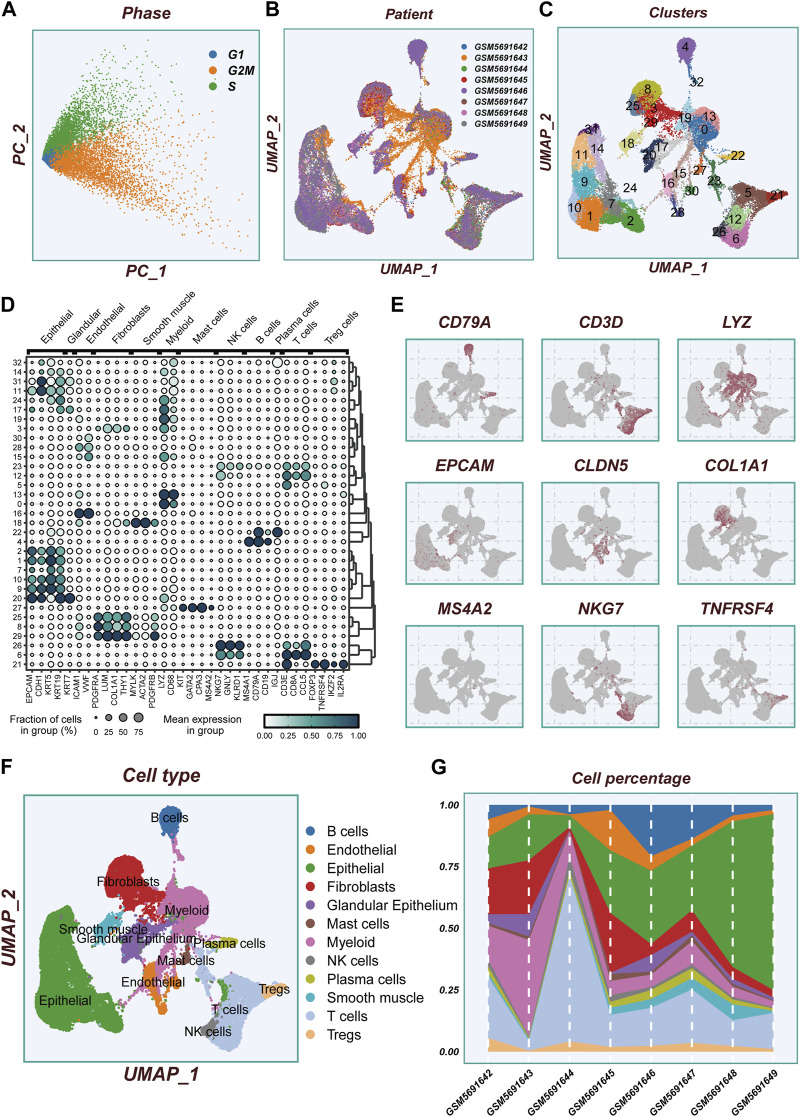
Flow chart of single cell analysis. **(A)**PCA dimensionality reduction clustering was performed according to the cell cycle related scores (G1, G2M, S-phase scores). **(B)** A tSNE plot showing the distribution of cell samples from different LUAD tissues. **(C)** A total of 33 cell subsets were separated by dimensionality reduction clustering. **(D)** Bubble plots showing the expression of marker genes corresponding to each cluster. **(E)** Multiple UMAP plots showing the expression of classic cell type marker genes. **(F)** A tSNE plot demonstrating the distribution of different cell types. **(G)** A histogram showing the variation of cell proportions between different samples.

Figure 1 displays the flow chart outlining the study. The results from the PCA analysis in Figure 2A indicate that there were no significant alterations observed in the cell cycle. This research involved a total of 8 samples, and Figure 2B demonstrates the distribution of the ESCC cells within each sample. It is worth noting that the samples were not significantly impacted by batch effects. Utilizing the uniform manifold approximation and projection (UMAP) dimensionality reduction algorithm, all cells were classified into 32 more detailed clusters, as depicted in Figure 2C. To showcase the expression of characteristic marker genes for each cell cluster, Figure 2D presents a bubble plot. Realistically, Figure 2E illustrates the distinctive metrics associated with different cell types. Additionally, Figure 2F presents the presence of 12 distinct cell types, including smooth muscle cells, T cells, and NK cells. The proportional representation of these 12 cell types in different samples is illustrated in Figure 2G.

### 3.2 Cell-cell interactions

**FIGURE 3:**
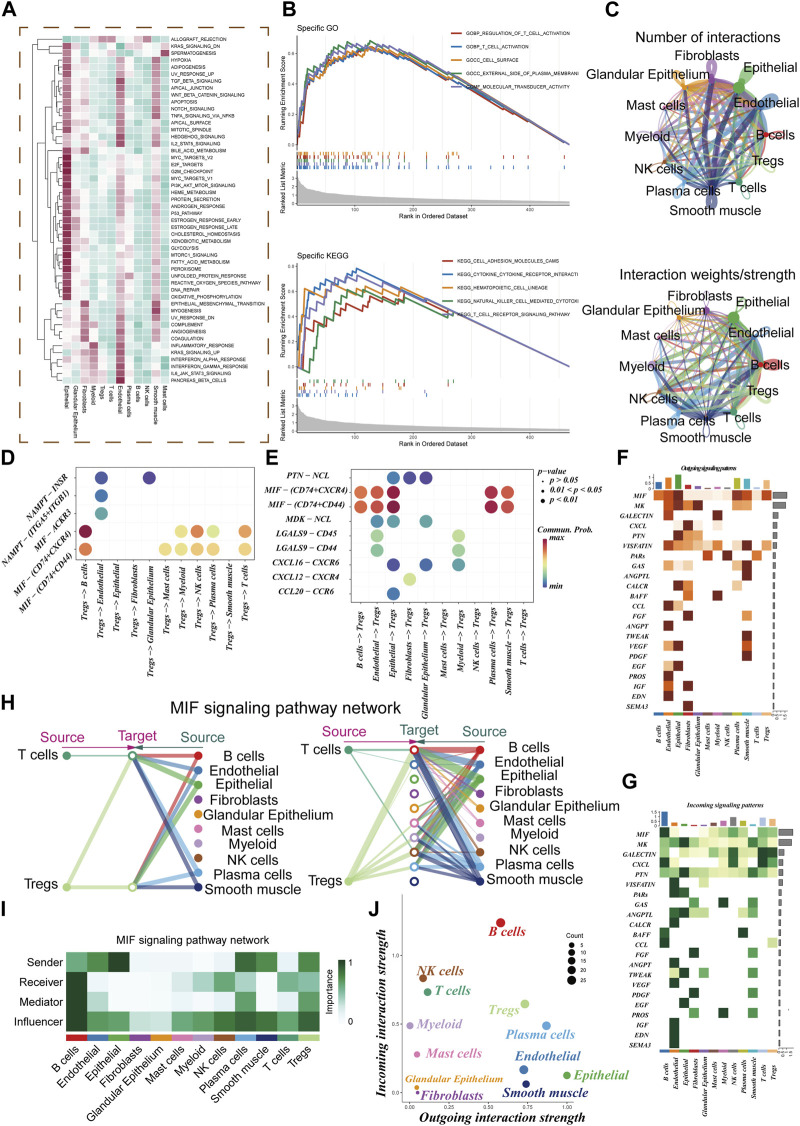
Cell-cell communication. **(A)** GSVA Enrichment Analysis: This panel depicts the enrichment of different cell subsets in the 50 hallmark gene sets. **(B)** GO and KEGG Enrichment Analysis: Here, we present the results of enrichment analysis focusing on marker genes in Treg cells. **(C)** Interaction Intensity: This section displays the number and intensity of interactions between different cell populations within the tumor microenvironment (TME). **(D,E)** Ligand-Receptor Pairs: These bubble plots highlight potential ligand-receptor pairs between Treg cells and other cell subpopulations in the TME. **(F,G)** Signaling Pathway Strength: Heatmaps visually represent the strength of outgoing and incoming signaling pathways across various cell subpopulations. **(H,I)** Role in MIF Signaling Pathway: This section outlines the roles of different cell populations in the MIF signaling pathway network within the TME. **(J)** Scatter Plot: Lastly, a scatter plot showcases the distribution of different cell populations concerning the intensity of outgoing and incoming signaling interactions.

The GSEA enrichment analysis visually depicted the enrichment of pathways in 12 distinct cell types (Figure 3A). The GO analysis revealed significant enrichment in essential pathways such as the regulation of T cell activation, cell surface function, and the external side of the plasma membrane. Additionally, the KEGG analysis demonstrated significant enrichment in pathways including cell adhesion molecules (cams), cytokine receptor interaction, and hematopoietic cell lineage (Figure 3B). Within the TME, robust cellular interactions were observed between Treg cells and several cellular components including B cells, epithelial cells, and NK cells (Figure 3C). These interactions play a pivotal role in immune responses and tumor development. The findings of this study uncovered that Treg cells can engage with B cells through the utilization of MIF-(CD74 +CXCR4) pairs (Figure 3D). Similarly, epithelial and plasma cells were found to communicate with Treg cells via the MIF-(CD74 +CXCR4) pairs (Figure 3E). This indicates that the MIF-(CD74 +CXCR4) axis serves as a critical signaling pathway facilitating crosstalk between Treg cells and various cell populations within the TME. Figures 3F, G illustrate the afferent and efferent signaling pathways of the 12 cell types, with MIF identified as the primary conduit for both types of signaling, playing a paramount role in intercellular communication. Figure 3H presents a hierarchical diagram showcasing the interactions between T cells, Treg cells, and other cell populations within the TME through typical MIF signaling pathways. The size of each circle corresponds to the number of cells in each cell group, while the thickness of the edges represents the probability of communication. Figures 3I, J provide evidence that Treg cells serve as both transmitters and receivers, actively facilitating communication with various neighboring cells.

### 3.3 Developing TAS model

**FIGURE 4:**
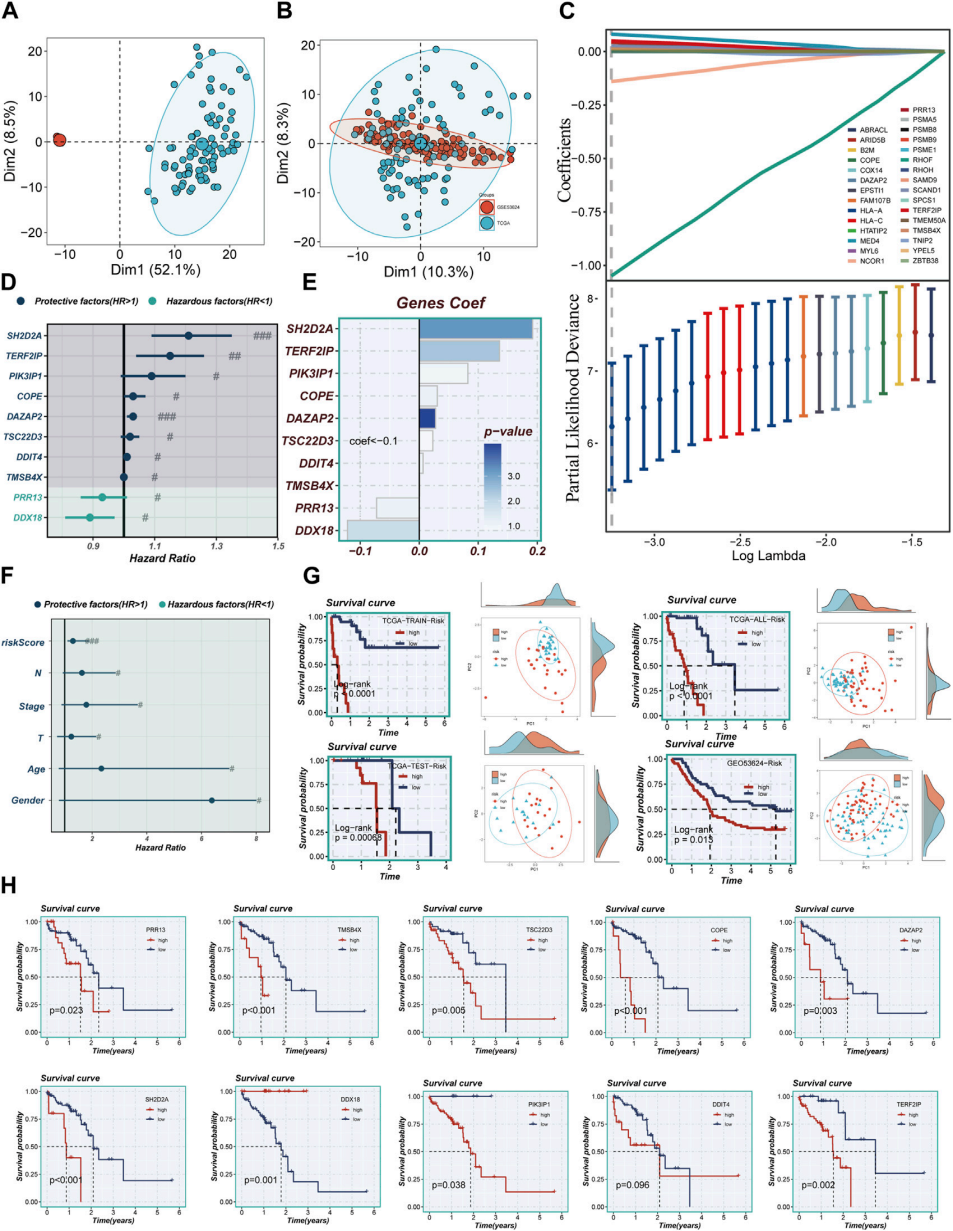
Construction of a stable risk model. **(A,B)** Sample distribution characteristics of two bulk RNA-seq cohorts before and after removal of batch effects. **(C)** LASSO regression screening for significant variables affecting prognosis. **(D)** Forest plot showing the results of multivariate COX analysis. **(E)** Distribution of coefficient values of model genes. **(F)** A forest plot was used to validate the constructed riskscore as an independent prognostic model. **(G)** The survival differences and PCA sample distribution of different risk groups in TCGA-TRAIN, TCGA-TEST, TCGA-ALL, and GEO53624, respectively, were presented. **(H)** Survival validation of the model genes. Note: # represents *p* < 0.05; ## represents *p* < 0.01; ### represents *p* < 0.001.

Figures 4A, B illustrate that the two datasets employed in the investigation, TCGA and GEO53624, underwent de-batching procedures to mitigate any batch effects. Notably, the TCGA cohort served as the reference for constructing the model. Utilizing Lasso and COX regression analyses, a comprehensive assessment led to the identification of ten marker genes associated with Treg cells, which were subsequently utilized to construct the Treg-associated signature (TAS). Among these genes, two were determined to be risk factors, while the remaining eight exhibited protective characteristics (Figures 4C, D). The corresponding coefficients of the ten model genes are visually presented in Figure 4E.

### 3.4 Assessing the performance of TAS

The calculation of the risk score for each individual patient was achieved through the multiplication of the expression levels of the genes in question by the corresponding coefficient. This mathematical modeling resulted in a quantifiable risk score, which was then utilized to categorize patients into two distinct groups: high-TAS group and low-TAS group. The division of these groups was determined by the median value of the calculated risk score. In Figure 4F, it was demonstrated that the risk score, much like other clinical characteristics, could serve as a prognostic risk factor for ESCC. Of particular note is the observation that patients classified in the high-risk group, as per the analysis of TCGA and GEO53624 cohorts, demonstrated a significantly poorer prognosis. Through PCA, it was demonstrated that the sample populations of the high- and low-TAS groups could be effectively segregated into two distinct clusters. This demonstrates not only the accuracy of the proposed model but also its robustness and stability (as presented in Figure 4G). Figure 4H further elucidates that the prognostic risk factors for ESCC patients are eight specific model genes: Proline Rich 13 (PRR13), SH2 Domain Containing 2A (SH2D2A), Thymosin Beta 4 X-Linked (TMSB4X), TSC22 Domain Family, Member 3 (TSC22D3), Phosphoinositide-3-Kinase Interacting Protein 1 (PIK3IP1), Coatomer Protein Complex Subunit Epsilon (COPE), TERF2 Interacting Protein (TERF2IP), and DAZ Associated Protein 2 (DAZAP2). Moreover, DEAD-Box Helicase 18 (DDX18) was identified as a prognostic protective factor for ESCC. Interestingly, the expression level of DNA Damage Inducible Transcript 4 (DDIT4) did not present a significant difference between the high- and low-TAS groups, indicating that its role in ESCC prognosis might be limited or non-significant.

### 3.5 Clinical correlation analysis

**FIGURE 5:**
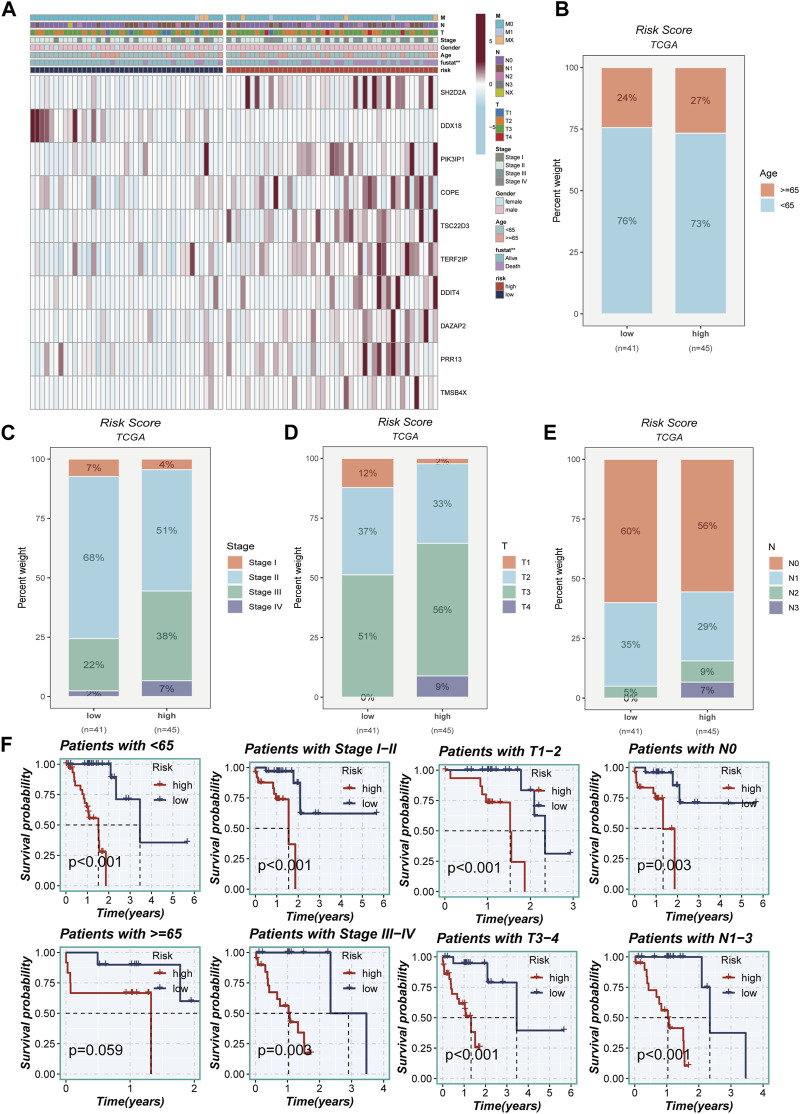
Clinical correlation analysis. **(A)** A heatmap was constructed by combining clinical features and model gene expression to demonstrate the distribution of clinical features and model genes in high- and low-risk groups. **(B–E)** Bar plots showing the proportion of age group, clinical stage, T-stage, and N-stage in the high- and low-risk groups. **(F)** Grouping based on clinical features to validate the impact of other clinical characteristics on the performance of the risk model.

Figure 5A portrays a captivating portrayal of the clinical features’ distribution within the high- and low-TAS groups, presented as a visually captivating heatmap. Within the high-TAS cohort, a noteworthy pattern emerges, revealing a correlation with advanced age, T-stage, N-stage, and clinical stage. These findings strongly indicate that patients belonging to this high-TAS group faced a relatively unfavorable prognosis, as highlighted in Figures 5B–E. To delve deeper into the prognostic potential of the riskScore, additional survival analyses were conducted. These analyses explored the predictive performance across various subgroups, including age subgroups, clinical staging subgroups, T-staging subgroups, and N-staging subgroups, unraveled in Figure 5F. Remarkably, the results consistently demonstrated that the riskScore maintained a robust ability to forecast outcomes within these distinct subgroups, further emphasizing its significance as a prognostic indicator.

### 3.6 Mutation landscape

**FIGURE 6:**
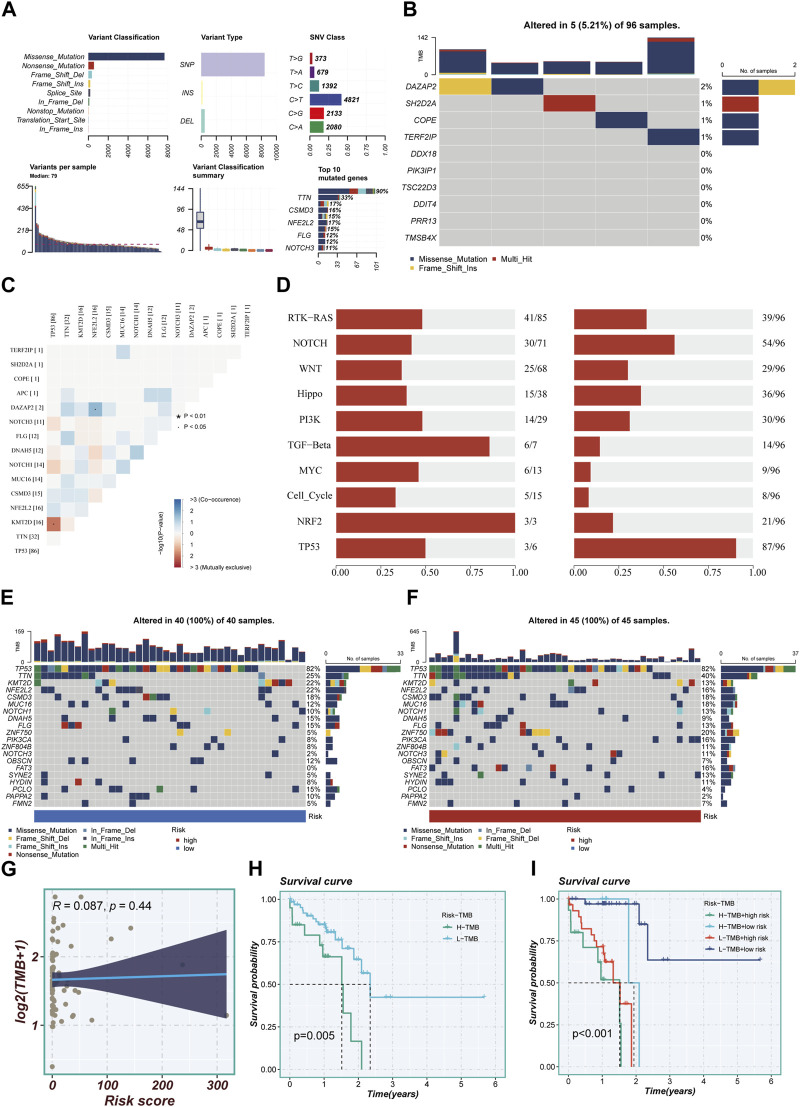
Mutation landscape in high- and low-risk groups. **(A)** Summary of mutational features in TCGA-ESCC samples. **(B)** An oncoplot of model genes in the TCGA-ESCC cohort. **(C)** Co-occurrence and mutual exclusivity relationships among the Top 15 ranked genes with mutations. **(D)** Enrichment status of 10 tumor-associated pathways. **(E,F)** mutation landscape of top 20 genes in high-risk and low-risk groups. **(G)** Scatter plot of correlation between risk scores and TMB. **(H)** Survival disparities between high and low mutation burden groups. **(I)** Survival curves showing the difference between survival among four subgroups (high-risk and high-mutation, high-risk and low-mutation, low-risk and high-mutation, low-risk and low-mutation).

The study meticulously investigated genetic alterations found in model-associated genes among the TCGA cohort of patients diagnosed with ESCC. The analysis unveiled that a noteworthy 5.21% of the ESCC patient population harbored mutations in a set of 10 pivotal genes. Of the top ten genetic aberrations found in the model-associated genes, TP53, a well-known tumor suppressor gene, exhibited the maximum incident rate, with an alarming 90% mutation frequency. The remaining genes in this set demonstrated mutation frequencies straddling a wide spectrum, from a moderate 11% to a substantial 33%, as depicted in Figures 6A, B. Figure 6C elucidates the interconnections among the top 15 mutated genes, showcasing KMT2D—a gene associated with histone modification—as the one exhibiting the strongest correlation with TP53. Figure 6D offers a comprehensive overview of the distribution of mutated genes across samples. The genetic alterations within high- and low-TAS groups were displayed separately, revealing that every patient within these groups experienced mutations. As with the broader cohort, TP53 retained its position as the gene with the highest mutation frequency, as demonstrated in Figures 6E, F. The Tumor Mutational Burden (TMB), a measure of the number of mutations carried by tumor cells, was found to be positively correlated with the risk score (Figure 6G). Notably, patients characterized by a high-TMB exhibited a significantly worse prognosis compared to their low-TMB counterparts, underscoring a clear divergence in outcomes (Figure 6H). The comparative analysis of survival curves across four distinct groups (High-TMB with high-risk, High-TMB with low-risk, Low-TMB with high-risk, and Low-TMB with low-risk) revealed a significant disparity (*p* < 0.001), further highlighting the crucial role of genetic mutations and TMB in determining the risk and survival of ESCC patients.

### 3.7 Enrichment analysis

**FIGURE 7:**
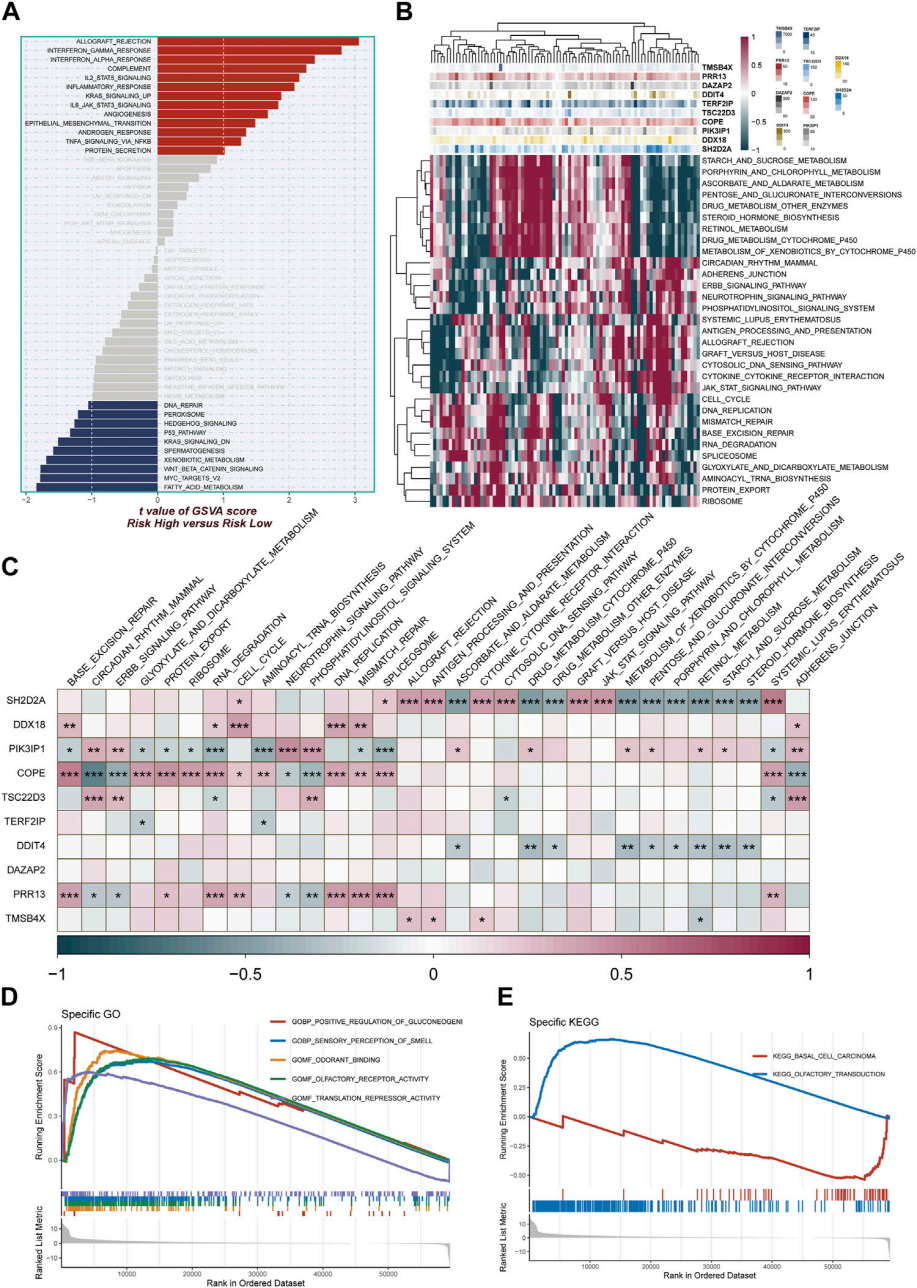
Enrichment pathways between different risk groups. **(A)** GSVA enrichment analysis demonstrates the enrichment of hallmark gene sets between different risk groups. **(B,C)** Heatmap and correlation plot depict the association between model genes and KEGG pathways. **(D,E)** GSEA enrichment analysis demonstrating the enrichment of GO and KEGG pathways between high- and low-risk groups.

The study harnessed GSVA to delve into pathway enrichment within the high-TAS group. Notably, our investigation unveiled significant enrichment in several pivotal pathways within this group, including angiogenesis, gamma interferon response, and IL2_STAT5 signaling (Figure 7A). These findings strongly suggest that these pathways may be either dysregulated or heightened in activity among individuals in the high-TAS group. Such dysregulation or increased activity could potentially contribute to their heightened susceptibility to specific medical conditions and, in turn, result in poorer prognostic outcomes. Figure 7B complements this by shedding light on the pathway enrichment of genes associated with each model. In order to further probe the intricate correlations between the 10 model genes and well-established pathways, we conducted a comprehensive correlation analysis. The results of this analysis are visually represented in Figure 7C, utilizing a heatmap to provide a clear overview of these relationships. Additionally, our study employed GSEA to shed light on the pathways affected. Remarkably, GSEA unveiled significant enrichment in pathways related to Positive regulation of gluconeogenesis, Translation repressor activity, and Odorant binding (Figures 7D, E). These enrichments may contribute to our understanding of why the high-TAS group experiences comparatively poorer prognostic outcomes, shedding light on potential mechanistic underpinnings of their condition.

### 3.8 Evaluating the level of immune infiltration

**FIGURE 8:**
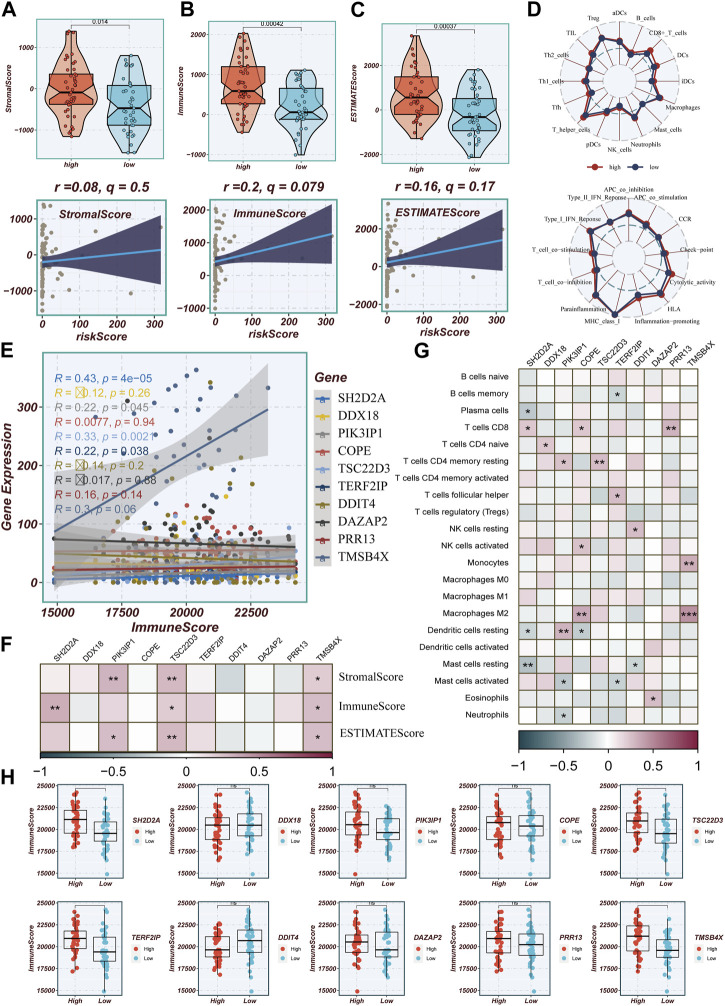
Evaluation of Immune Infiltration between High and Low-Risk Groups. **(A–C)** Box plots depicting differences in stromal score, immune score, and estimate score between high and low-risk groups. Scatter plots illustrating the correlation of risk scores with stromal score, immune score, and estimate score. **(D)** Assessment of immune cell infiltration and immune-related pathways differences between high and low-risk groups using the ssGSEA algorithm. **(E)** Correlation between model genes and immune scores. **(F)** Correlation heatmap illustrating the relationship between model genes and stromal score, immune score, and total score. **(G)** Evaluation of the correlation between model genes and immune cell infiltration using the CIBERSORT algorithm. **(H)** Differences in immune scores between groups stratified by high and low expression of model genes.

Differences in immune infiltration between high- and low-TAS groups in TCGA-ESCC were assessed by using data obtained from 7 immune infiltration algorithms in TIMER. The results showed that the high-TAS group had relatively high levels of immune infiltration, while the low-TAS group had relatively low levels of immune infiltration (Supplementary Figure S1A). The heatmap demonstrated the correlation analysis of model-related genes with common components in TME (Supplementary Figure S1B). In addition, this study validated the immune infiltration levels in different risk groups using the ESTIMATE method. This method estimates the immune infiltration and the stromal fraction in the interstitium of tumor tissues. It was found that the immune score was lower in the low-TAS group compared to the high-TAS group. Spearman’s correlation analysis showed that the risk score was positively correlated with immune infiltration (Figures 8A–C). ssGSEA analysis showed that the high-risk group had a higher abundance of infiltrating immune cells, especially CD8+_T cells and T_helper cells (Figure 8D). Figure 8E shows that the level of immune infiltration correlated with model gene expression, with the highest correlation with TMSB4X. Figure 8F shows the correlation of stromal score, immune score, and ESTIMAT score with the model-related genes, and it is obvious that the correlation with TMSB4X, SH2D2A, PIK3IP1, and TSC22D3 is higher. The heatmap demonstrates the analysis of the correlation of the 10 model genes with the various types of cells in the TME (Figure 8G), with most of them being positively correlated. Figure 8H shows the comparison of immune infiltration between the high and low expression groups of each model gene, in which there was a significant difference in the level of immune infiltration between the high and low expression groups of genes such as TMSB4X, SH2D2A, TERF2IP, and TSC22D3.

### 3.9 Immunotherapy and chemotherapy drugs

**FIGURE 9:**
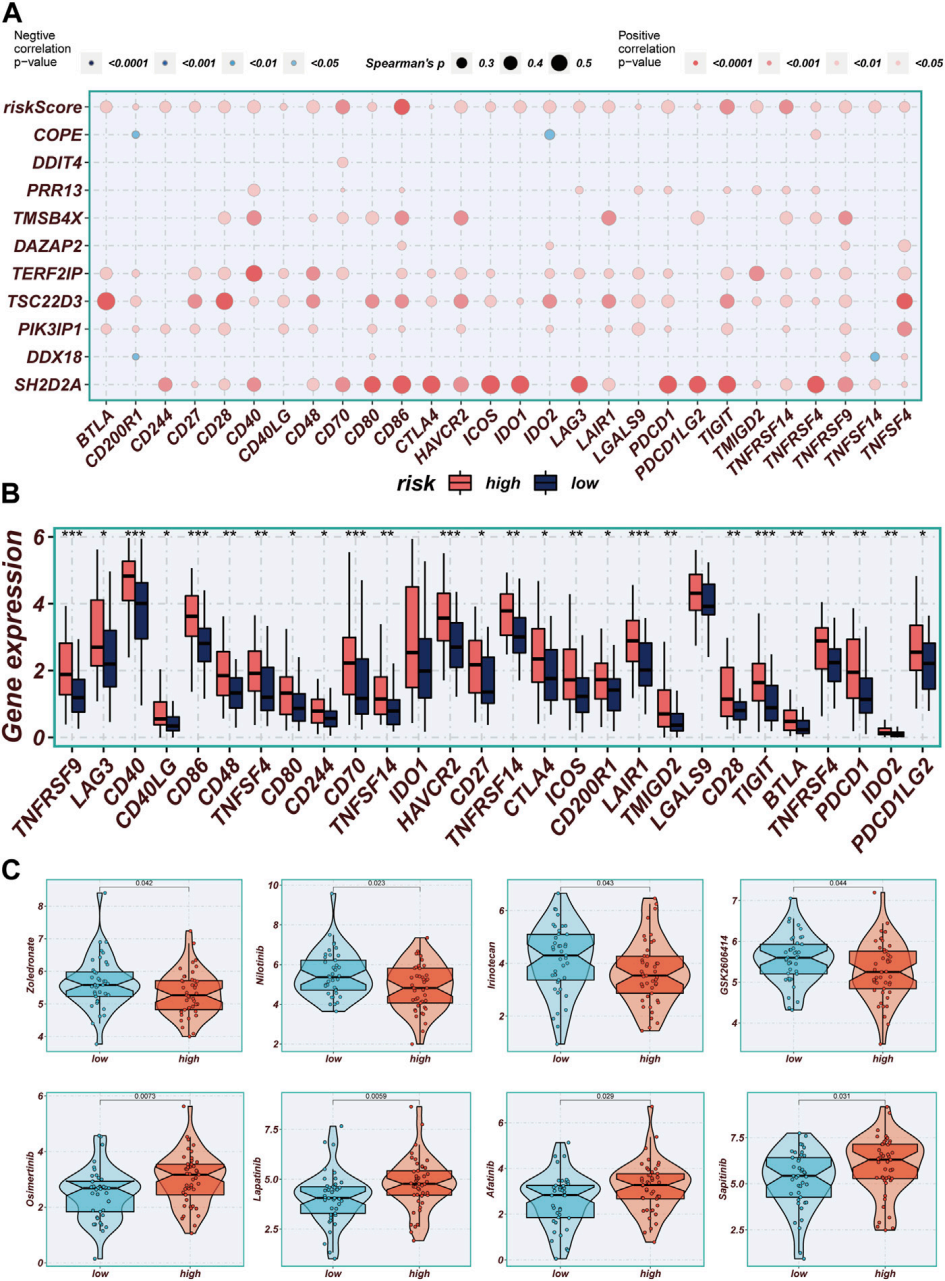
Immune checkpoint and immunotherapy analysis. **(A)** Correlation scatter plots showing the correlation between model genes and risk scores and immune checkpoint expression. **(B)** Boxplots showing the difference in immune checkpoint expression between high- and low-risk groups. **(C)** Boxplots demonstrating the possible sensitivity of chemotherapeutic agents between different risk groups.

Correlations between modeled genes, risk scores, and ICGs were depicted using bubble plots (Figure 9A). Blue color indicates negative correlation and orange color indicates positive correlation. Interestingly, there was a positive correlation between risk scores and all ICGs, whereas the expression levels of model genes were positively correlated with the vast majority of ICGs and negatively correlated with individual ICGs, including CD200R1, IDO2, and TNFSF14. In this study, we analyzed the relationship between mature ICGs and risk scores in the TCGA cohort. It was found that the expression of all ICGs-related genes was higher in the high-risk group than in the low-TAS group, such as CD28, CD40LG and TNFRSF9 (Figure 9B). Drug sensitivity analysis showed that Zoledronate, Nilotinib, Irinotecan, and GSK2606414 might enhance the efficacy in the high-TAS group, while Osimertinib, Lapatinib, Afatinib, and Sapitinib showed higher sensitivity in the low-risk group (Figure 9C). Re-validation in the IMvigor210 cohort revealed that the risk scores in the CR/PR group were significantly higher than those in the SD/PD group, indicating that immunotherapy was more effective in the high-risk group and that patients in the high-risk group had a shorter survival than those in the low-risk group, suggesting that the present prediction model is also applicable to bladder cancer (Supplementary Figure S2A, B).

### 3.10 Performing experimental validation

**FIGURE 10:**
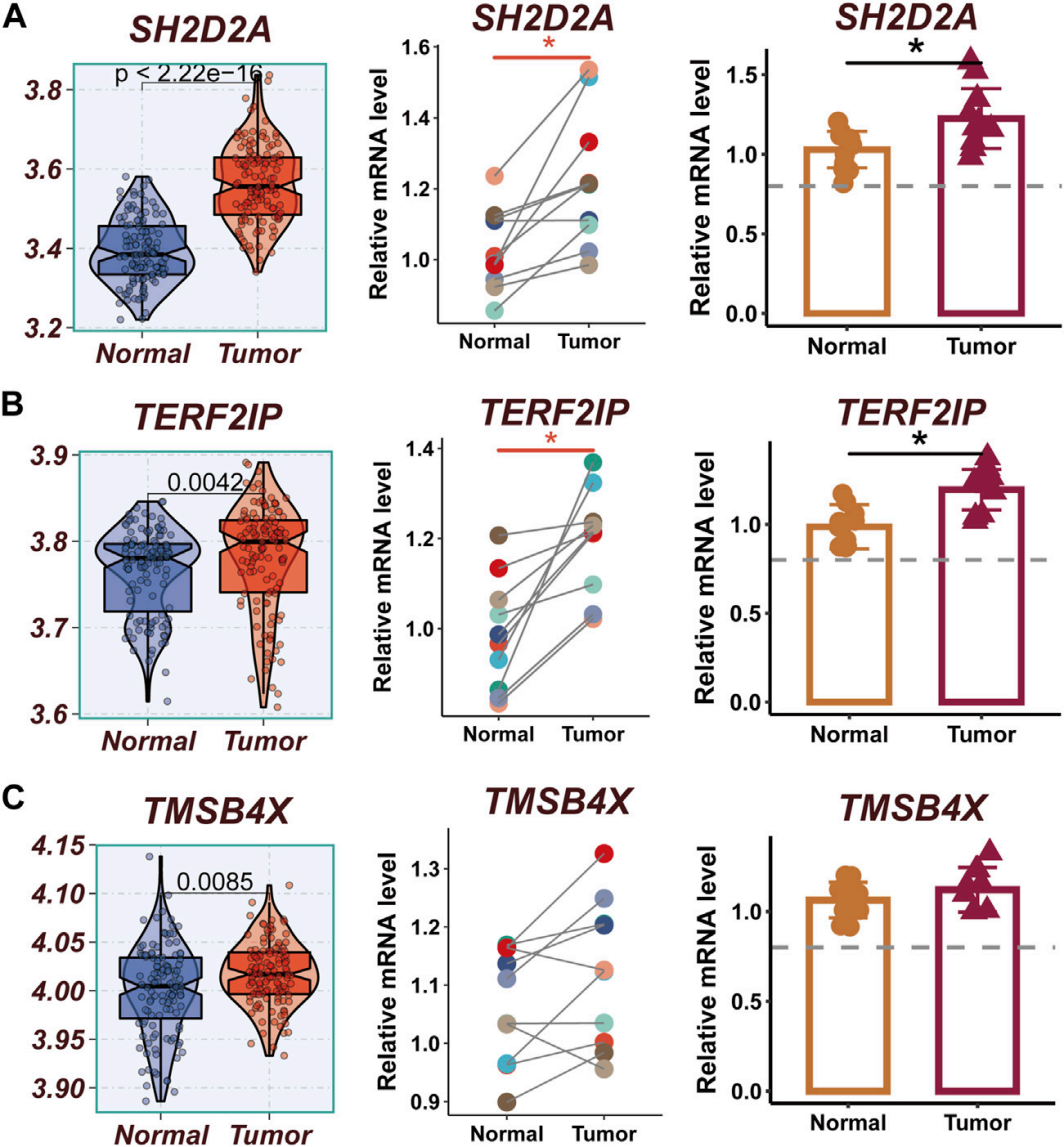
Experimental validation of model gene. **(A)** Boxplots showing the differential expression of SH2D2A between tumor and normal tissues in TCGA-ESCC; Relative expression of LTB gene in 10 pairs of cancer and paracancer samples, respectively. **(B)** Boxplots showing the differential expression of TERF2IP between tumor and normal tissues in TCGA-ESCC; Relative expression of TERF2IP gene in 10 pairs of cancer and paraneoplastic samples, respectively. **(C)** Boxplots showing the differential expression of TMSB4X between tumor and normal tissues in TCGA-ESCC; Relative expression of TMSB4X gene in 10 pairs of cancer and paraneoplastic samples, respectively.

The expression levels of SH2D2A, TERF2IP, and TMSB4X were significantly different between normal and tumor samples of TCGA, while other model genes were not significantly different. To verify this finding, qRT-PCR was performed using surgically resected tumor tissue and normal esophageal tissue. The results showed that the expression of SH2D2A and TERF2IP genes was significantly upregulated in the tumor tissues, whereas the expression of TMSB4X, although also elevated, was not statistically different (Figures 10A–C).

## 4 Discussion

Esophageal cancer is a highly aggressive malignancy with a significant global burden, characterized by high mortality rates and unfavorable prognoses (Xia et al., 2022). ESCC represents the predominant histological subtype, comprising approximately 95% of all esophageal cancer cases (Zou et al., 2018). The immune system plays a pivotal role in the pathogenesis of cancer, exerting its influence by modulating the body’s specific anti-tumor immune response capacity, thereby potentially impeding tumor cell dissemination and metastasis (Zimmermann and Curtis, 2019; Gong et al., 2023).

Treg cells have been identified as active participants in tumor progression and development, exerting their influence through the suppression of anti-tumor immunity and facilitation of immune evasion (Corral-J et al., 2021). Within TME, various factors contribute to the resistance observed in immune checkpoint blocker therapy, with Treg cells playing a pivotal role in immunosuppression within the TME (Pitt et al., 2016). The accumulation of Treg cells within the TME hampers the immune response against tumors and is considered a critical mechanism by which tumors evade immune surveillance (Wang et al., 2019a). Enhanced infiltration of CCR4+ Treg cells has been associated with unfavorable prognoses in different cancer types, including pancreatic ductal adenocarcinoma, prostate cancer, and colorectal cancer (Grage-Griebenow et al., 2014; Wang et al., 2019b; Watanabe et al., 2019).

Treg cells contribute to immune evasion primarily through the production of various soluble factors, including Interleukin-10(IL-10) and transforming growth factor-β(TGF-β). TGF-β is an immunosuppressive cytokine that plays a crucial role in the activation and sustenance of Forkhead box protein P3(FOXP3) expression and Treg cells function. The regulation of the TGF-β signaling pathway directly impacts the development, stability, and functionality of Treg cells. Additionally, multiple cellular components within TME, such as lymphocytes, fibroblasts (including cancer-associated fibroblasts or CAFs), bone marrow-derived inflammatory cells, blood vessels, and extracellular matrix, can influence the migration, generation, expansion, and activity of Treg cells in the TME (Joyce and Fearon, 2015). For instance, a specific subset of CAFs known as CAF-S1 fibroblasts can attract Treg cells through CXCL12 secretion, retain Treg cells by upregulating OX40L, PD-L2, and JAM2 expression, and induce the differentiation of T-lymphocytes into Treg cells by increasing the expression of B7H3, CD73, and DPP4. These mechanisms collectively contribute to the establishment of an immunosuppressive microenvironment (Costa et al., 2018). Hence, it is essential to investigate the involvement of Treg cells in ESCC to facilitate the development of efficacious immunotherapy strategies. Targeting Treg cells or modulating their suppressive capabilities holds promise as a potential avenue to augment anti-tumor immune responses and ameliorate the prognosis of ESCC patients.

Single-cell analysis offers unparalleled resolution for studying intratumor heterogeneity, cellular differentiation trajectories, and intercellular communication, thus offering promising applications. By analyzing cell clustering in the scRNA-seq dataset, we identified genes that are specifically expressed in tumor cells, thus shifting the focus from the comparison of tumor to normal tissue observed in previous database analyses to exploring the distinctions between tumor cells themselves (Huang et al., 2020).

In this study, we harnessed scRNA-seq data to pinpoint crucial marker genes intricately associated with Treg cells. Leveraging the power of integrated multiple bulk RNA-seq datasets, we meticulously crafted a prognostic signature comprising a set of ten genes. Subsequently, we employed this signature to calculate risk scores, effectively stratifying patients diagnosed with ESCC into two distinct groups: high-TAS and low-TAS. Through a series of rigorous survival analyses conducted across multiple cohorts, including the TCGA test cohort, TCGA training cohort, TCGA whole cohort, and GEO53624 cohort, a consistent and compelling pattern emerged. Patients within the low-TAS group consistently exhibited a more favorable prognosis, as evidenced by statistical significance (*p* < 0.05). To gain deeper insights into the underlying biological mechanisms at play, we conducted a comprehensive functional enrichment analysis. This analysis unveiled the significant enrichment of the TAS in specific pathways, shedding light on why individuals in the high-TAS group might experience comparatively poorer prognostic outcomes. These pathways included those associated with the positive regulation of gluconeogenesis, translation repressor activity, and odorant binding. It is worth noting that prior investigations have illuminated the role of miR-145 as a potent suppressor of PLCE1 expression, a susceptibility gene known to promote ESCC development. miR-145 achieves this inhibitory effect by suppressing the translation of PLCE1, ultimately curbing the proliferation, migration, and metastasis of esophageal cancer cells. This insight contributes to our understanding of the molecular mechanisms underlying the differential prognosis observed between high- and low-TAS groups in ESCC patients (Cui et al., 2016).

A comprehensive examination of the immune cells infiltrating the tumor could shed light on the mechanisms underlying cancer immune evasion, presenting an opportunity to develop innovative therapeutic strategies (Zhang and Zhang, 2020). Through an assessment of immune cell infiltration in the high- and low-TAS groups, this study revealed a higher abundance of immune cell infiltration in the high-TAS group compared to the low-TAS group. Previous research has established a correlation between the expression levels of immune checkpoint genes and the effectiveness of immunotherapy (Ahluwalia et al., 2021). Analysis of the variations in immune checkpoint gene expression between the high- and low-TAS groups suggests that alternative immune checkpoints could be targeted for treatments in the low-TAS group. Therapeutic interventions focused on TME have emerged as a promising approach in cancer treatment, given the critical role of the TME in influencing tumor progression and response to conventional therapies (Bejarano et al., 2021). Notably, TME scores exhibited a statistically significant distinction in immune scores between the low- and high-TAS groups. This finding implies that patients in the low-TAS group may exhibit heightened responsiveness to immunotherapy.

The SH2D2A gene encodes a T cell-specific adaptor protein, known as T cell-specific adaptor (TSAd), that regulates early T cell activation (Kolltveit et al., 2008). It is involved in the interaction between vascular endothelial growth factor receptor 2 (VEGFR2) and c-Rous sarcoma (c-Src), leading to c-Src activation and promotion of tumor vascular permeability (Matsumoto et al., 2005; Sun et al., 2012). TERF2IP is a component of the protective protein complex responsible for safeguarding telomeric DNA and maintaining chromosomal stability (de Lange, 2005). It has been implicated in tumorigenesis, progression, and chemoresistance in various human cancers. Overexpression of TERF2IP has been observed in breast, gastric, non-small cell lung, condylomatous lymphoma, multiple melanoma, colorectal, and renal cell carcinomas (Panero et al., 2014; Panero et al., 2016; Pal et al., 2017; Xiao et al., 2017; Anu et al., 2020; Bhari et al., 2021). However, studies investigating the role of TERF2IP in ESCC are currently lacking. TMSB4X, also known as thymosin beta-4, is a member of the thymosin beta family. It acts as an actin chelating protein, playing a crucial role in regulating actin polymerization. TMSB4X is involved in intracellular signaling and has been found to be overexpressed in colorectal, lung, gastric, pancreatic, head and neck squamous, and squamous cell carcinomas (Chu et al., 2019). Ma et al. identified a prognostic signature comprising 16 genes, including TMSB4X, which accurately predicts the prognosis of ESCC patients (Ma and Luo, 2022).

In our investigation, we substantiated the heightened expression levels of SH2D2A and TERF2IP in tumor tissues based on our analysis of clinical surgical resection samples. Moreover, we noted a noteworthy increase in the expression of TMSB4X within tumor tissues, although statistical significance was not firmly established. It is imperative to acknowledge that the study’s limitations stem from the relatively small size of our validation cohort. Consequently, future endeavors should prioritize validation with larger sample sizes to fortify these findings. Additionally, our study underscores the essential need for further experimental investigations to delve into the biological functions and underlying mechanisms of action of the candidate genes we identified. By doing so, we can attain a more comprehensive understanding of their roles in the context of ESCC. To summarize, the prognostic signature developed in this study holds significant promise as a valuable tool for prognostic predictions in ESCC patients. This signature also opens up exciting possibilities for the integration of immunotherapeutic approaches into the clinical management of these patients.

## 5 Declarations

### 5.1 Ethical approval and consent to participate

The Ethics Committee of the First Affiliated Hospital of Nanjing Medical University granted approval for all human experiments conducted within this study. Prior to their participation, all subjects provided informed consent for inclusion. The study was meticulously executed in strict adherence to the principles outlined in the Declaration of Helsinki and received official endorsement from the Ethics Committee of the First Affiliated Hospital of Nanjing Medical University (protocol code No. 2SRFA-005; dated 27 February 2019).

## Data Availability

The original contributions presented in the study are included in the article/Supplementary Material, further inquiries can be directed to the corresponding authors.
