# Supplementary figures and images for: Deciphering Treg cell roles in esophageal squamous cell carcinoma: a comprehensive prognostic and immunotherapeutic analysis
Source: Front Mol Biosci. 2023 Sep 28;10:1277530. doi: 10.3389/fmolb.2023.1277530 (PMC10568469; doi:10.3389/fmolb.2023.1277530)

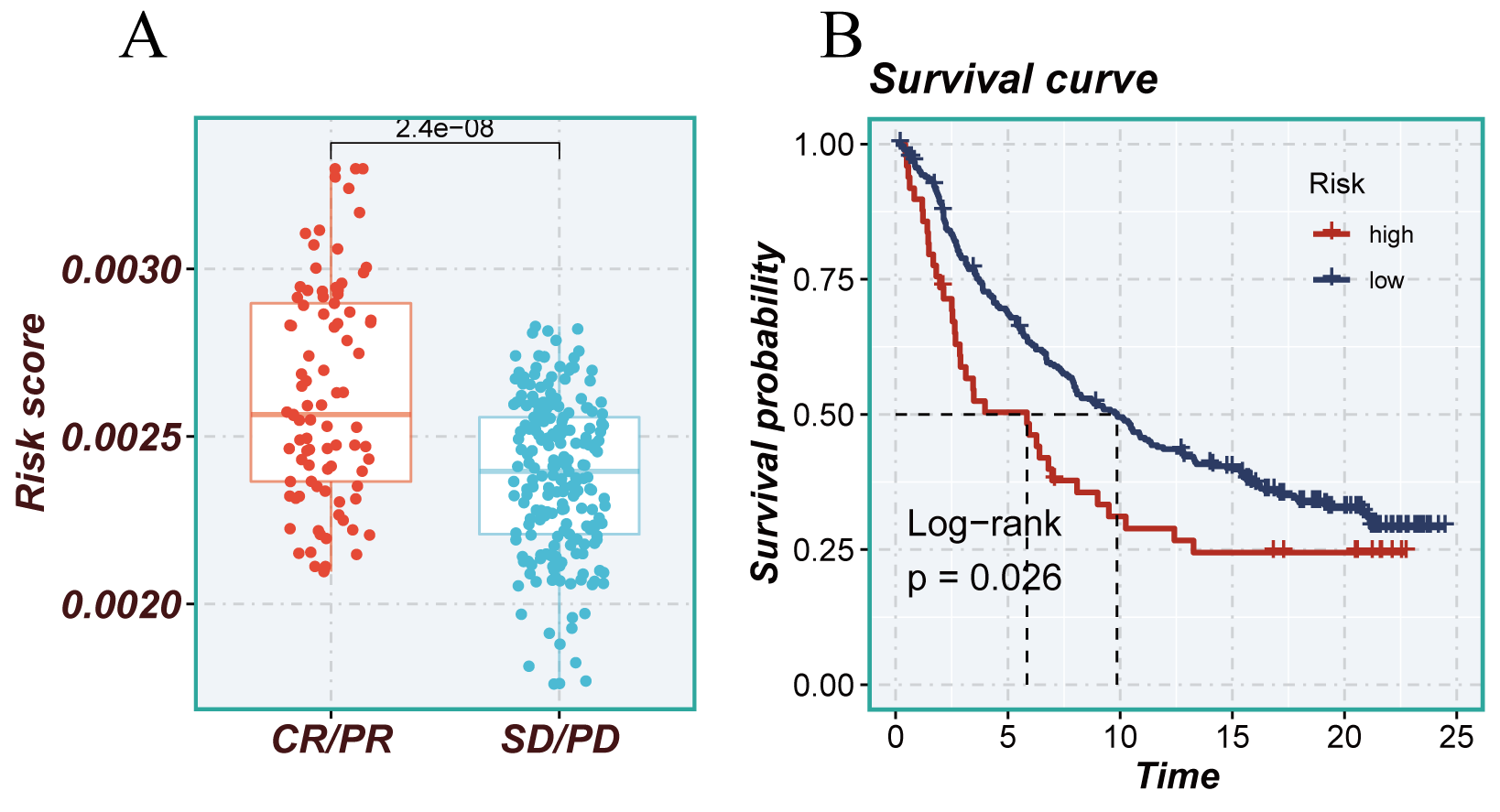

Supplement: Supplementary file 1 [file Image2.TIF]

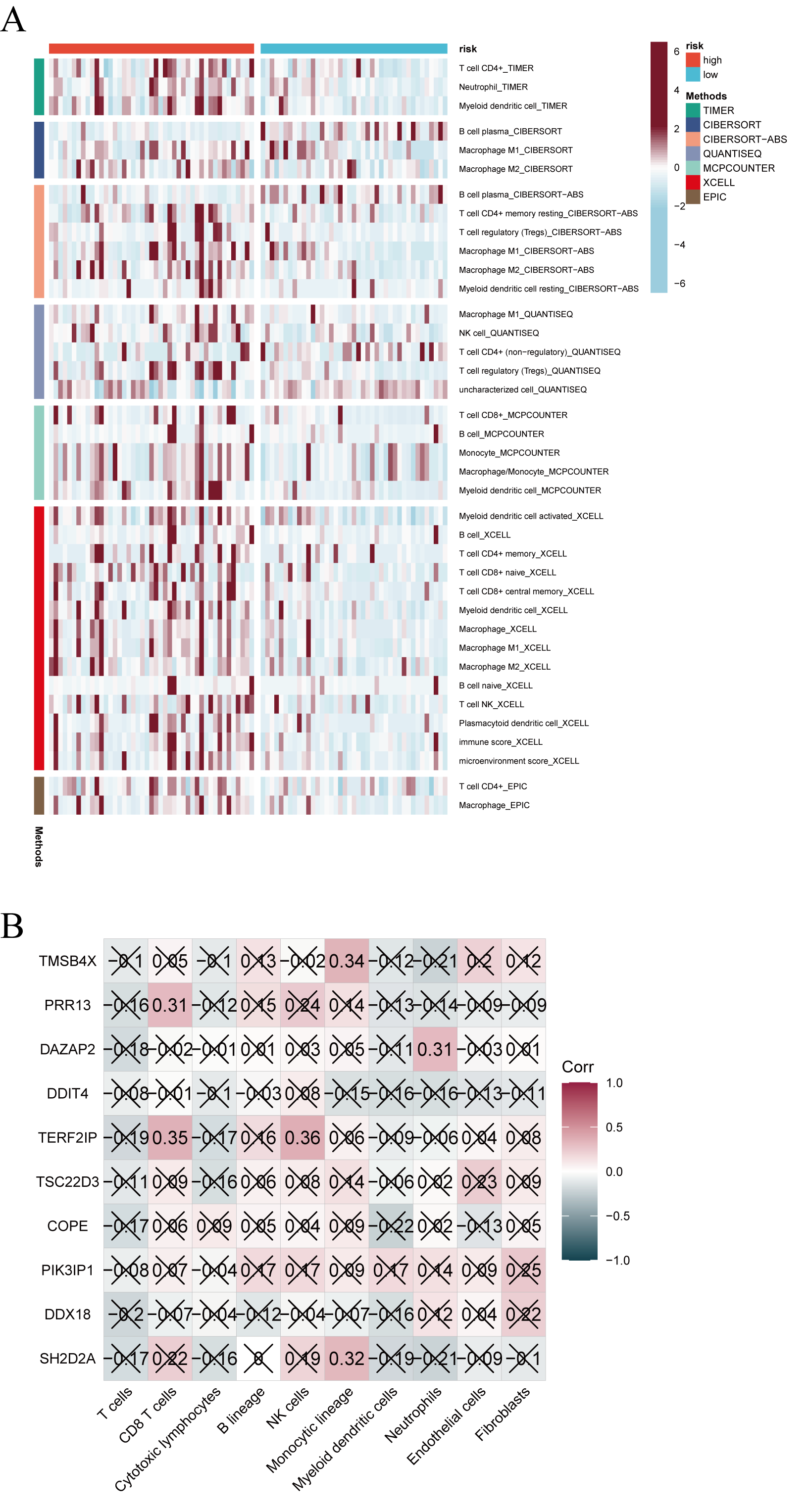

Supplement: Supplementary file 2 [file Image1.TIF]
